# Adrenomedullin 2 increases cardiac sympathetic nerve activity in parallel to heart rate in normal conscious sheep

**DOI:** 10.14814/phy2.14096

**Published:** 2019-05-23

**Authors:** Christopher J. Charles, David L. Jardine, Miriam T. Rademaker, A. Mark Richards

**Affiliations:** ^1^ Department of Medicine Christchurch Heart Institute University of Otago Christchurch Christchurch New Zealand

**Keywords:** Arterial pressure, cardiac output, sympathetic nervous system

## Abstract

Both adrenomedullin 2 (AM2) and sympathetic nerve activity (SNA) have been shown to be involved in regulating cardiovascular activity, but whether any interaction between these two systems exists remains to be determined. In this study, we examine the effects of intravenous AM2 infusions on SNA directed toward the heart (cardiac SNA (CSNA)) in healthy sheep studied in the conscious state. In response to AM2, arterial pressure was reduced (*P* = 0.005) with both heart rate (*P* < 0.001) and cardiac output (*P* < 0.001) increased compared with vehicle control response. CSNA burst frequency (bursts/min) and burst area/min both increased during infusion of AM2 (both *P* < 0.001). However, correcting CSNA indices for concurrent heart rate changes resulted in CSNA burst incidence (bursts/100 beats) and burst area incidence (area/100 beats) being not significantly different between AM2 and control treatments. There were no significant differences demonstrated in plasma epinephrine or norepinephrine levels between the two study days. In conclusion, AM2 administered systemically to normal conscious sheep increases both CSNA and heart rate. However, correction for heart rate responses abrogates the rise in CSNA. It remains unclear whether AM2's primary effect is to act via the central nervous system to directly stimulate CSNA with resultant increase in heart rate, or to induce a rise in heart rate by other mechanisms.

## Introduction

Sympathetic nerve activity (SNA) is a key contributor to cardiovascular regulation both in normal health and disease. The adrenomedullin (AM) family of peptides have also been shown to play a role pressure/volume homeostasis and in controlling vascular tone (Ishimitsu et al. 2006). Both SNA and the AMs are activated in cardiovascular disease. The AM family of peptides, including AM and adrenomedullin 2 (AM2, otherwise known as intermedin), signals through a common set of receptor complexes consisting of the calcitonin receptor‐like receptor (CLR) linked with one of three receptor activity‐modifying proteins (RAMPs). AM has been shown to activate CLR/RAMP2 and CLR/RAMP3 preferentially, whereas AM2 binds nonselectively to all three CLR/RAMP combinations (Roh et al. 2004). The fact that the different AM peptides show different profiles of receptor activation is suggestive of differences in down‐stream bioactivity. Also of note, the AM receptor/RAMP combinations show variability in their regional distribution (RAMP2 > > RAMP1 = RAMP3 in the heart; RAMP1 > RAMP2 in the aorta; RAMP3 > RAMP2 in the kidneys) (Nagae et al. 2000) and regulation (Nagae et al. 2000; Totsune et al. 2001) of the three RAMP isoforms. Thus, while the vasoactive activity of AM and AM2 shows similarity, the different selectivity for the receptor/RAMP complexes suggest some differences in bioactivity are likely.

Bioactivity displayed by AM2 is in general similar to that reported to date for AM, including sustained and potent falls in arterial pressure with concomitant increases in cardiac output and heart rate. These effects are consistently observed in several species including rats (Roh et al. 2004; Takei et al. 2004; Fujisawa et al. 2006, 2007) and sheep (Charles et al. 2006). One point of difference between AM and AM2 is response of plasma aldosterone which rises in healthy sheep in response to intravenous (IV) infusion of AM2 (Charles et al. 2006). In contrast, IV infusions of AM do not increase plasma aldosterone levels in sheep and humans despite concomitant activation of plasma renin activity (Charles et al. 1997; Lainchbury et al. 1997, 1999; Rademaker et al. 1997). This is one key difference between the bioactivity of the two peptides identified to date.

We have previously shown that AM, which induces falls in arterial pressure with concomitant rises in cardiac output and heart rate, increases cardiac sympathetic nerve activity (CSNA) and heart rate to a greater degree than nitroprusside infusions titrated to match the fall in arterial pressure in normal conscious sheep (Charles et al. 2005). There are several reports of the response of regional SNA to AM2. Microinjection of AM2 into the paraventricular nucleus (PVN) attenuates the cardiac afferent sympathetic reflex and reduces renal sympathetic nerve activity (RSNA) in coronary artery ligated rats (Gan et al. 2014). In contrast, systemic administration of AM2 increased RSNA in conscious intact rats, with effects attenuated, but not abolished, by sino‐aortic denervation (Fujisawa et al. 2006). Tissue receptor density may also vary across different organs such as heart, kidney, and blood vessels which may in part explain differences in tissue response. Furthermore, the response to a number of different vasodilators (both exogenous and endogenous) can show significant regional selectivity of sympathetic activation (Ninomiya et al. 1971; Pagani et al. 1974). Thus, there is a need to measure efferent sympathetic traffic to the specific organs of interest. With the interaction between systemic AM2 and CSNA currently unknown, we set out to examine the effects of IV infusion of AM2 on CSNA and hemodynamics in normal conscious sheep.

## Materials and Methods

The study protocol was approved by Animal Ethics Committee of the University of Otago‐Christchurch. Coopworth ewes (*n* = 8 obtained from Lincoln University Research Farm, Christchurch, New Zealand) were housed under standard husbandry conditions receiving standard sheep diet of pelleted food supplemented with lucerne hay giving a daily intake of 75 mmol sodium and 150 mmol potassium. General anesthesia was induced by 17 mg/kg IV thiopentone sodium, sheep intubated and ventilated with maintenance anesthesia of isoflurane, nitrous oxide, and oxygen. Sheep were prepared for conscious CSNA recording as previously described (Jardine et al. 2002). Briefly, a left lateral thoracotomy was performed for placement of five stainless steel needle electrodes into the thoracic cardiac nerves which were glued in place. The connected leads were glued and sutured to the mediastinum before being exteriorized through the chest wall alongside the scapula. In addition, a 16G angiocath cannula was placed in a carotid artery to directly measure arterial pressure and heart rate during subsequent infusions studies, polyethylene catheters were inserted into the jugular veins for subsequent peptide infusions and to obtain serial blood samples and a Swan‐Ganz catheter floated into the pulmonary artery via the jugular vein for subsequent thermodilution cardiac output recordings. In accordance with our approved ethics protocol, animals received prophylactic and postoperative antibiotics (cephazolin) and analgesia including caprofen, intercostal nerve block with mixture of lignocaine and marcaine and temgesic. Sheep were left to recover postoperatively for at least 4 days prior to infusion studies.

CSNA recordings were obtained from selected pairs of electrodes (best combinations tested during postoperative recovery) by means of an active probe connected to a preamplifier (DAM‐80, World Precision Instruments). After amplification (×1000) the raw signal was filtered (300–3000 Hz) and integrated using 100 msec as the time constant. Digital conversion of the integrated nerve signal was performed using in‐house software with a sampling rate of 200 Hz, and postganglionic efferent sympathetic activity was confirmed by the following criteria: (1) bursts were synchronized to arterial diastole, (2) CSNA was suppressed during IV infusion of hexamethonium (2 mg/kg over 2 h) performed 3 days postoperatively, and (3) CSNA burst area was inversely proportional to diastolic arterial pressure during baroreflex testing with IV bolus of nitroprusside and phenyephrine (performed >60 min prior to commencement of AM2 infusion). A signal‐to‐noise ratio of >2 was the threshold required for nerve bursts to be included in analysis. Once the best pair of electrodes was selected on the first day, the same pair were used for all subsequent CSNA recording. CSNA was measured in four different ways; burst frequency (bursts/minute); burst incidence (bursts/100 beats); burst area (total area under all qualifying bursts/minute); and burst area/100 beats.

Heart rate and CSNA baroreflex slopes were determined during the hypotensive response following intravenous bolus injections of nitroprusside (150 *μ*g). Heart rate and CSNA indices were measured over consecutive 10 beat packages for 3–5 baseline periods and then for the full duration of the hypotensive response induced by nitroprusside. Linear regression of mean arterial pressure (MAP) to heart rate and CSNA allowed calculation of the respective rises in response to a fall of 8 mmHg MAP from baseline levels (pressure matched for the AM2‐induced fall in MAP seen below).

Sheep were studied twice, receiving in balanced random order vehicle control (40 mL haemaccel) and IV AM2 (5.5 pmol/kg/min or 33 ng/kg/min) infused for a duration of 120 min. Arterial pressure recordings were performed from 30 min prior to infusions till 60 min postinfusion. Mean arterial pressure (MAP) and heart rate were digitally integrated in five minute recording blocks at preset time points throughout the protocol by means of our in‐house computerized data acquisition/analysis system. Thermodilution cardiac output was measured (>3 results ± 10%) at the same time points as MAP. Venous blood was taken into EDTA tubes (chilled) at the same time points. After centrifugation, plasma was stored at ‐80°C prior to assay for plasma epinephrine and norepinephrine (Goldstein et al. 1981).

### Statistics

Results are expressed as mean ± SEM. Primary analysis was by two‐way analysis of variance using time as the repeated measure. This determined whether a significant time × treatment interaction existed between control and AM2 study days. Post hoc protected fisher's least significant difference (LSD) tests determined individual time points that were significantly different between control and AM2 data. *P* < 0.05 was the threshold for statistical significance.

## Results

Data collection was complete and the studies proceeded without mishap. Baseline CSNA burst frequency showed variation between study days (control 46.3–60.1 and AM2 range 27.5–85.9 bursts/min), as did baseline burst incidence (control range 43.8–72.8 and AM2 range 33.2–89.0 bursts/100 beats). Thus, both of these indices are presented as percentage change from baseline. As arbitrary units are used to calculate the CSNA burst area these data are presented as percentage change from baseline.

**Figure 1:**
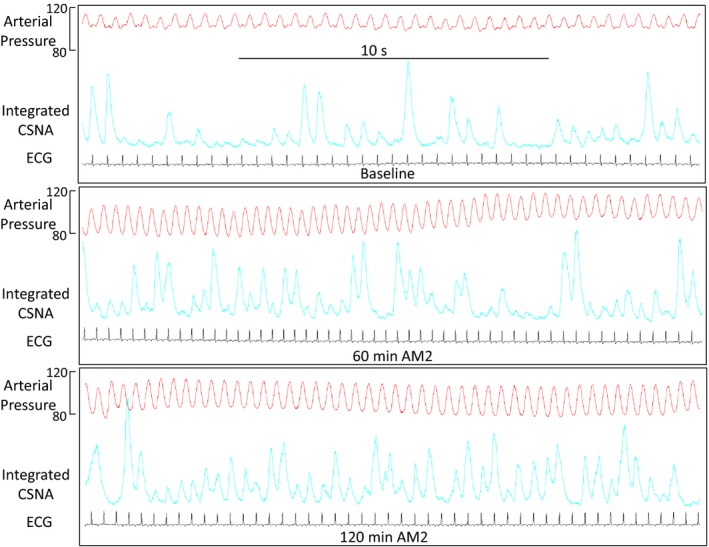
Sample recordings from a representative sheep of arterial pressure, integrated cardiac sympathetic nerve activity (CSNA), and ECG at baseline and after 60 and 120 min of adrenomedullin 2 (AM2) infused at 33 ng/kg/min for 120 min.

Figure 1 shows a representative trace of ECG, arterial pressure, and integrated CSNA at baseline and then 60 and 120 min after commencement of AM2 infusion. All peaks of integrated CSNA burst activity are synchronized to arterial pressure diastole. AM2 induces a fall in arterial pressure and an increase in heart rate associated with a rise in CSNA burst frequency and burst area (a combination of burst magnitude and frequency).

**Figure 2:**
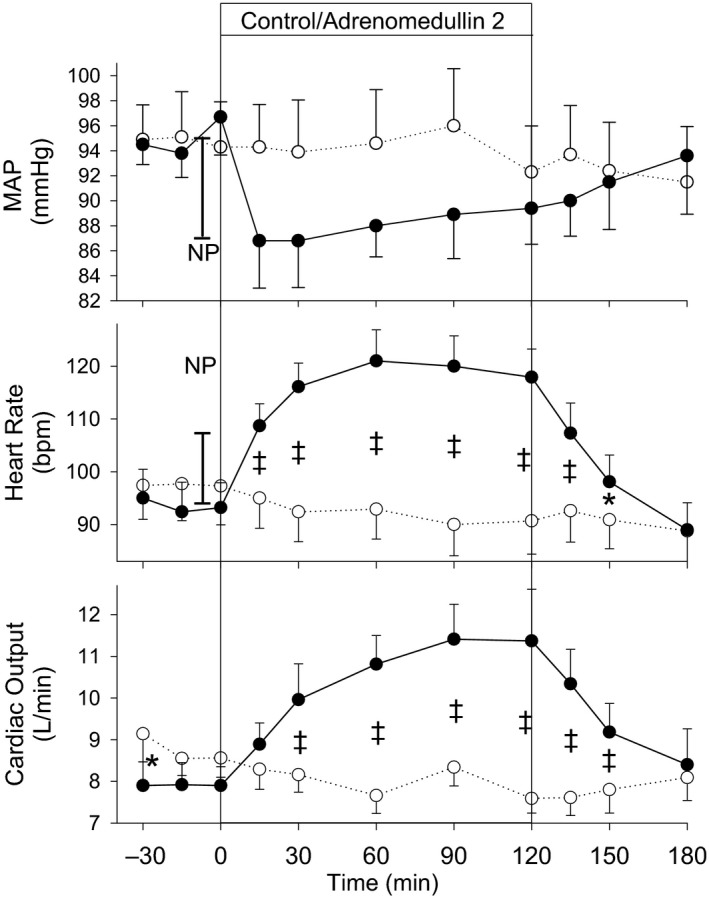
Mean arterial pressure (MAP), heart rate, and cardiac output response to intravenous infusions of adrenomedullin 2 (●) or vehicle control (○) in 8 normal conscious sheep. Values shown are mean ± SEM. Significant differences were observed for MAP (*P* = 0.005), heart rate (*P* < 0.001), and cardiac output (*P* < 0.001). Individual time points significantly different from time‐matched control (Fisher's protected LSD from two‐way ANOVA) are indicated by *P < 0.05 and ^&ddagger;^ *P* < 0.001. Vertical bars marked NP represent the baroreflex rise in heart rate in response to an 8 mmHg fall in MAP during a nitroprusside bolus administered prior to the infusions.

Compared with time‐matched control, MAP fell promptly during AM2 infusion (*P* = 0.005) (Fig. 2), with the maximum fall in MAP (8 mmHg) apparent by 15–30 min and pressures remaining approximately 4–6 mmHg lower for most of the AM2 infusion period. Heart rate was increased in response to AM2 (*P* < 0.001) compared with control. Heart rate was maintained at least 30 bpm above time‐matched control from 30 min after commencement of AM2 and remained elevated for the duration of infusion, not returning to vehicle control levels until 60 min after cessation of AM2. By comparison, an 8 mmHg fall in MAP in response to nitroprusside bolus administered prior to the start of AM2 infusion resulted in a 13.3 ± 2.89 bpm rise from baseline in heart rate (vertical bar noted on Fig. 2). Cardiac output showed a similar time course, with levels increased for the duration of AM2 infusion and for a further 60 min following cessation of AM2 compared with control (*P* < 0.001).

**Figure 3:**
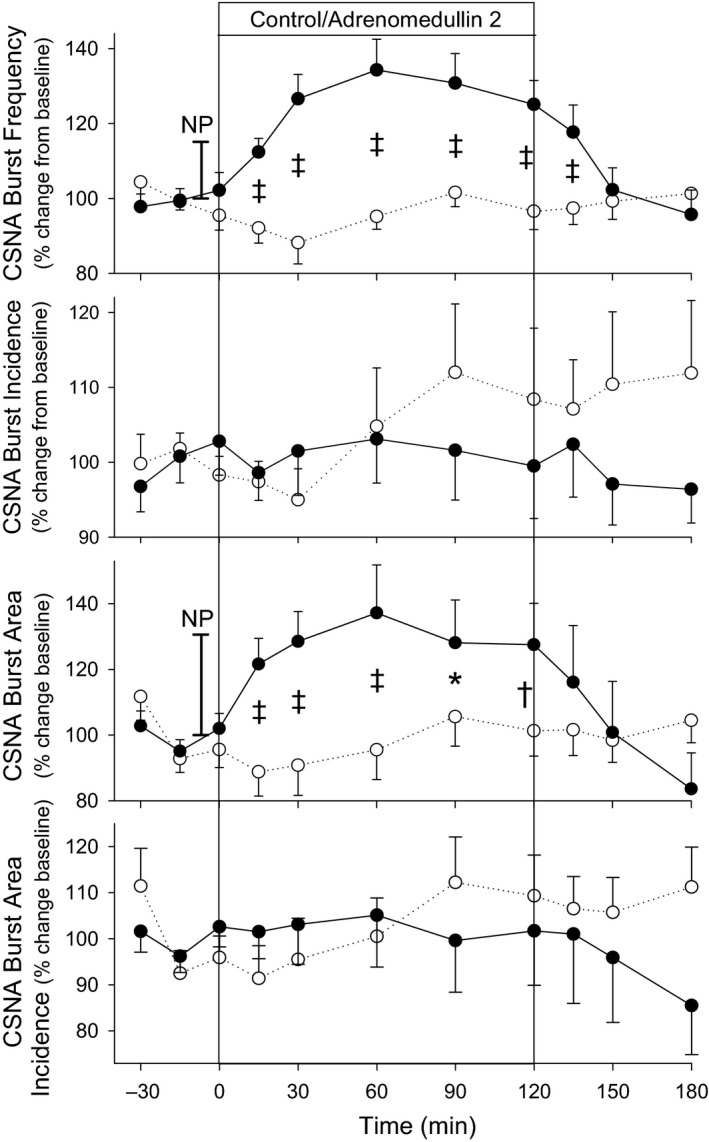
CSNA response to intravenous infusions of adrenomedullin 2 (●) or vehicle control (○) in 8 normal conscious sheep. Values shown are mean ± SEM. Significant differences were observed for CSNA burst frequency (bursts/min; *P* < 0.001) and CSNA burst area (*P* < 0.001). Individual time points significantly different from time‐matched control (Fisher's protected LSD from two‐way ANOVA) are indicated by *P < 0.05, ^†^ *P* < 0.01, and ^&ddagger;^ *P* < 0.001. Vertical bars marked NP represent the baroreflex rise in CSNA burst frequency and burst area in response to an 8 mmHg fall in MAP during a nitroprusside bolus administered prior to the infusions.

CSNA burst frequency (burst/min) rose during infusion of AM2 (*P* < 0.001) compared with control being 25–35% higher throughout AM2 infusion, with the time course of changes very similar to that seen for heart rate changes (Fig. 3). By comparison, an 8 mmHg fall in MAP in response to nitroprusside bolus administered prior to the start of AM2 infusion resulted in a 15.13 ± 4.65 percentage rise from baseline in CSNA burst frequency (vertical bar noted on Fig. 3). In contrast, CSNA burst incidence, which normalizes for any heart rate changes (bursts/100 beats), showed no significant difference between AM2 and control (Fig. 3). Similarly, CSNA burst area/min also increased in response to AM2 (*P* < 0.001) in the range of 27–37% higher during AM2 compared with control (Fig. 3). This compares with a rise of 30.6 ± 8.81 percentage rise from baseline in CSNA burst area (vertical bar noted on Fig. 3) in response to an 8 mmHg fall in MAP following nitroprusside bolus administered prior to the start of AM2 infusion. Again, with normalization for concurrent heart rate changes, CSNA burst area/100 beats showed no significant difference between AM2 and control (Fig. 3).

**Figure 4:**
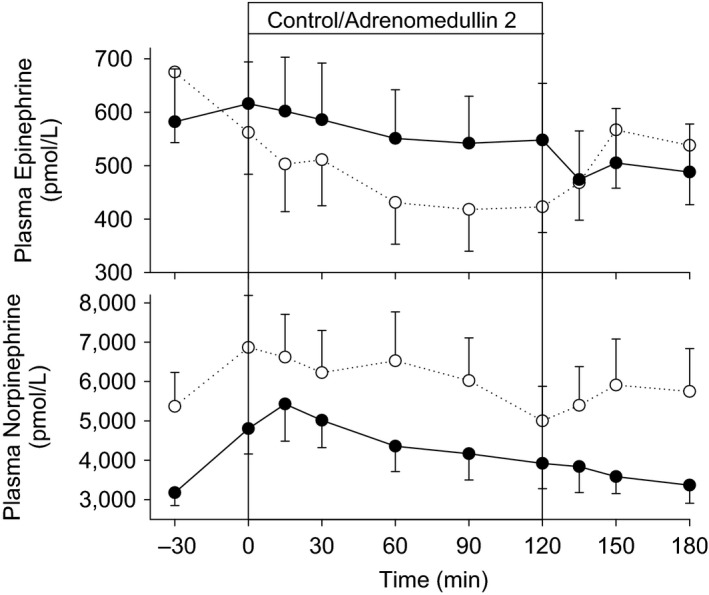
Plasma norepinephrine and epinephrine response to intravenous infusions of adrenomedullin 2 (●) or vehicle control (○) in normal conscious 8 sheep. Values shown are mean ± SEM.

As seen in Figure 4, neither plasma epinephrine nor norepinephrine showed any significant changes between the control and AM2 study days.

## Discussion

There are no previous reports of the effect of AM2 on CSNA and previously reported bioactivity for AM2 has limited mechanistic explanation. It remains unclear whether the direct vasodilatory actions of AM2 are responsible for its other biological actions. Therefore, in this study we have examined the effects of AM2 on CSNA with concurrent measurement of arterial pressure, heart rate, and cardiac output in healthy conscious sheep. IV infusion of AM2 induces concomitant falls in MAP and rises in heart rate and cardiac output. CSNA burst frequency and burst area measured per minute mirror the increase in heart rate but normalization of CSNA for heart rate changes to give burst area/100 beats and burst incidence resulted in no significant increase in these CSNA indices.

AM2 was first described in (Roh et al. 2004; Takei et al. 2004) with an early report showing that when infused systemically AM2 increased RSNA in conscious rats (Fujisawa et al. 2006). This rise in RSNA was in the setting of hypotension, but both heart rate and RSNA increases were greater with AM2 than seen with pressure‐matched nitroprusside and baroreceptor (sino‐aortic) denervation which only partially attenuated the increases in heart rate and RSNA. Taken together, this indicates that AM2 may play a key role in directly regulating the sympathetic nervous system. Since that initial publication, the only other reports of AM2 interaction with SNA is with regional administration of the peptide to various central nervous system (CNS) nuclei. Microinjection of AM2 into the nucleus tractus solitarii (NTS) raised MAP and RSNA through a pathway mediated by AM1/2 receptors, protein kinase A (PKA), and cyclic adenosine monophosphate (Li et al. 2013). The hypertensive effect of intracerebroventricular AM2 was abolished by α‐adrenoceptor antagonists (Taylor et al. 2005), suggesting that AM2 increases arterial pressure at least in part by increasing SNA in the CNS. In contrast to those results, when AM2 was administered to the PVN, both RSNA and MAP were reduced via an AM1/2 receptor and (NO) oxide synthase‐mediated mechanism (Zhou et al. 2014). These effects were greater in two‐kidney, one‐clip (2K1C) hypertensive rats compared with sham control normotensive rats. In another study, AM2 injected into the PVN attenuated the cardiac sympathetic afferent reflex and reduced RSNA in coronary artery ligated rats (Gan et al. 2014). Taken together, central administration of AM2 has different actions dependent upon the site of injection. As with many other peptides, central effects on SNA cannot be extrapolated to predict the SNA effects of systemically administered peptide.

The only previous report of systemically administered AM2 on directly measured SNA is that of Fujisawa et al. 2006, which showed AM2 raised RSNA. We had previously shown that systemic AM2 had no significant effect on circulating levels of epinephrine or norepinephrine (Charles et al. 2006), a finding repeated in this study. Of note, plasma catecholamines are a relatively blunt tool for assessing SNA and only measure global changes without any organ specific changes. Also of note, there can be regional selectivity of sympathetic reflex activity with traffic directed to different organs and tissues varying greatly (Ninomiya et al. 1971; Pagani et al. 1974). Thus, there is a need to measure efferent sympathetic traffic to the specific relevant regions. This is the first study to examine effects of AM2 infusion on CSNA. Of note, CSNA plays a key role in the pathophysiology of cardiovascular disease, thus, unravelling interactions between neurohumoral factors such as AM2 and SNA may prove pivotal in understanding cardiac function in both normal physiology and pathophysiology.

AM2 induces a clear rise in CSNA as measured by the indices measured per minute. Comparison of the CSNA burst frequency rise in response to nitroprusside bolus administered prior to commencement of AM2 infusion shows that in response to an 8 mmHg fall in MAP (matched to that induced by AM2) that the rise in CSNA burst frequency heart rate was approximately 40–60% less than that induced by pressure‐matched AM2, namely 15 versus 25–35 bursts/min. No formal statistical comparison was performed on these CSNA responses as, despite matched degree of hypotension, the data was not time‐matched and the nitroprusside was determined in response to bolus administration, whereas the AM2 was during continuous infusion. Nonetheless, the higher CSNA rise with AM2 compared with nitroprusside could suggest that, rather than just a baroreflex‐mediated increase in CSNA burst frequency, it is possible that there may have been some contribution from direct central actions to the increase in CSNA. However, arguing against this it is important to note that, with correction for concomitant heart rate, CSNA burst incidence (bursts/100 beats) and burst area/100 beats were not significantly raised above vehicle control levels. However, it is important to note that, with correction for concomitant heart rate, burst incidence and burst area/100 beats were not significantly increased compared to the vehicle control data. It has been known for several decades that CSNA bursts demonstrate diastolic entrainment (McAllen and Malpas 1997). Thus, we could postulate that when heart rate is increased that CSNA burst frequency and burst area/minute might rise proportionately. It is not clear from data presented here whether AM2 primarily stimulates CSNA by means of a direct action on heart rate (CNS mediated) or whether AM2 raised heart rate by another mechanism (including vagal withdrawal). Follow‐up studies are required to measure CSNA during AM2 administration with concomitant control of heart rate (e.g., by pacing or muscarinic blockers). Alternatively measurement of both vagal activity and CSNA at the same time could also elucidate key mechanisms. With respect to direct CNS mechanisms of systemically administered AM2, there are no previous reports of AM2 crossing the blood–brain barrier, but of note, the structurally related AM does cross the blood–brain barrier (Kastin et al. 2001). Alternatively, AM2 may activate receptors within the circumventricular organs which then signal to key brain nuclei as per the mechanisms invoked for many other vasoactive hormones which work both systemically and in the CNS (such as angiotensin II).

The hemodynamic actions of AM2 demonstrated in this and previous (Charles et al. 2006) studies are comparable to actions previously described for AM (Charles et al. 1997). Thus, it seems probable that the mechanisms underlying the hemodyamic actions are the same for AM and AM2. AM2's effects on cardiac output are probably multifactorial and could be secondary to changes in cardiac afterload and/or preload, increased efferent CSNA (baro‐reflex mediated), a direct positive inotropic action or via changes in heart rate. Early reports from in vivo studies (Charles et al. 2006; Fujisawa et al. 2007) and in isolated rat hearts (Yang et al. 2005) suggest that AM2 may increase contractility. AM2 increases cardiac contraction by a direct mechanism to increase intracellular Ca^2+^ in PKC and PKA‐dependent pathways in murine ventricular myocytes (Dong et al. 2006). It also has a positive inotropic effect in anesthetized pigs (Grossini et al. 2009). However, there have also been reports of AM2 decreasing cardiac contractility (pP/dt) in isolated rat hearts (Pan et al. 2005). Munzel et al. (2011) reported opposite effects in cell culture, where cultured cardiomyocytes demonstrated positive inotropic effects of AM2, versus isolated hearts, where AM2 decreased contractility via a NO‐dependent mechanism. Clearly, additional studies are required to determine under which experimental conditions AM2 has direct inotropic actions. In vitro studies have shown AM2 to have a direct vasodilatory action on aortic, carotid, coronary, and supramesenteric rings (Kobayashi et al. 2004; Pan et al. 2005). Furthermore, we have previously reported that the hypotensive effects of AM2 were greater on diastolic compared to systolic pressures, again indicating a direct vasodilator effect which may be the primary mechanism underlying this action of the peptide.

The rise in heart rate observed in both this and previous studies may represent a primary baro‐reflex response to falls in MAP. However, sino‐aortic denervation in rats only partially reduces the heart rate (and RSNA) increase to AM2, and the rise in heart rate was higher than that seen with pressor‐matched nitroprusside (Fujisawa et al. 2006). Of note in this study, heart rate response was more long‐lived than the hypotension, with heart rate remaining 30 bpm higher throughout the AM2 infusion, whereas MAP was no longer significantly reduced compared to control by end of infusion. Furthermore, heart rate did not return to control levels till 60 min postinfusion, 60–90 min after MAP returned to control. Comparison of the heart rate rise to nitroprusside bolus administered prior to commencement of AM2 infusion shows that in response to an 8 mmHg fall in MAP (i.e., matched to that induced by AM2) that the rise in heart rate was only approximately 40–50% of that induced by pressure‐matched AM2, namely 13 versus 30 bpm (again, no formal statistical comparison performed as above). However, this result is in agreement with studies performed in rats (Fujisawa et al. 2006) in which heart rate response was greater with AM2 than nitroprusside and also similar to that previously shown for AM in sheep (Charles et al. 2005). Thus, it is clear that AM2 induced more than just the expected baroreflex increase in heart rate. It is likely that AM2 has some direct chronotropic actions contributing to the sustained rise in heart rate, also reflected in the time course of cardiac output response. It is also possible that AM2 may act via the CNS to induce vagolytic effects contributing to exaggerated heart rate rise as has been previously described for AM (Parkes and May 1997). Measurement of both vagal activity and CSNA in future studies could help elucidate key mechanisms. However, there was neither direct or indirect measurement of vagal activity made in this study. Clearly, the effects of AM2 on cardiovascular homeostasis, including heart rate and cardiac output responses, are multifactorial.

To our knowledge there are no validated assays for measurement of AM2 in circulation. Therefore, plasma levels of AM2 in normal physiology or pathophysiology are not known. Given this lack of background information, we designed this study to use a dose and duration of AM2 that would give physiologically relevant perturbations in arterial pressure and other key hemodynamic indices but that would not invoke excessive baroreceptor activation. We were familiar with hemodynamic response to infused doses of AM2 in conscious sheep at 10 and 100 ng/kg/min having previously published this study (Charles et al. 2006). Thus, we postulated that the intermediate dose of 33 ng/kg/min used in this study would give some (but not excessive) fall in arterial pressure over a 2 h period with concomitant changes in heart rate and cardiac output. The dose and duration used in this study was also identical to that we previously reported for effect of AM on CSNA (Charles et al. 2005). It is yet to be determined whether this represents a physiological or pharmacological dose.

A limitation of this study of systemic infusion of AM2 is that it acts on all tissues and organs of the body and consequently it is difficult to determine whether the cardiovascular responses obtained are direct or indirect. Future studies investigating more direct actions of AM2 on CSNA by local infusion of the compound onto the stellate ganglion or key autonomic ganglia in the CNS such as the NTS or RVLM would help to define central actions on CSNA.

In conclusion, this is the first report of the effect of AM2 administration on CSNA in normal conscious sheep. We demonstrated that AM2 induces an increase in both CSNA indices measured per minute (burst frequency and burst area/min) in parallel to a rise in heart rate. When CSNA indices were corrected for the heart rate response, CSNA burst incidence and burst area/100 beats were no longer statistically raised compared with control. It remains unclear whether AM2's primary effect is to act via the central nervous system to directly stimulate CSNA with resultant increase in heart rate, or to induce a rise in heart rate by other mechanisms. Follow‐up studies are required to measure CSNA during AM2 administration with concomitant control of heart rate or alternatively measurement of both vagal activity and CSNA could also elucidate key mechanisms.

## Conflict of Interest

None declared.
